# Evaluation of forensic and anthropological potential of D9S1120 in Mestizos and Amerindian populations from Mexico

**DOI:** 10.3325/cmj.2012.53.423

**Published:** 2012-10

**Authors:** Héctor Rangel-Villalobos, Viviana M Sánchez-Gutiérrez, Miriam Botello-Ruiz, Joel Salazar-Flores, Gabriela Martínez-Cortés, José F Muñoz-Valle, Christopher Phillips

**Affiliations:** 1 Institute of Molecular Genetics Research, University of Guadalajara, Ocotlán, Jalisco, Mexico; 2 Department of Molecular Biology and Genomics, University of Guadalajara, Guadalajara, Jalisco, Mexico; 3 Forensic Genetics Unit, Institute of Legal Medicine, University of Santiago de Compostela, Santiago de Compostela, Spain; Rangel-Villalobos et al: D9S1120 in Mestizos and Amerindian populations from Mexico

## Abstract

**Aim:**

To carry out a deeper forensic and anthropological evaluation of the short tandem repeat (STR) D9S1120 in five Mestizo populations and eight Amerindian groups from Mexico.

**Methods:**

We amplified the STR D9S1120 based on primers and conditions described by Phillips et al, followed by capillary electrophoresis in the genetic analyzer ABI Prism 310. Genotypes were analyzed with the GeneMapper ID software. In each population we estimated statistical parameters of forensic importance and Hardy-Weinberg equilibrium. Heterozygosity and F_ST_-values were compared with those previously obtained with nine STRs of the Combined DNA Index System (CODIS-STRs).

**Results:**

Amerindian and Mestizo populations showed high frequencies of the allele 9 and 16, respectively. Population structure analysis (AMOVA) showed a significant differentiation between Amerindian groups (F_ST_ = 2.81%; *P* < 0.0001), larger than between Mestizos (F_ST_ = 0.44%; *P* = 0.187). D9S1120 showed less genetic diversity but better population differentiation estimates than CODIS-STRs between Amerindian groups and between Amerindians and Mestizos, but not between Mestizo groups.

**Conclusion:**

This study evaluated the ability of D9S1120 to be used for human identification purposes and demonstrated its anthropological potential to differentiate Mestizos and Amerindian populations.

Sample analysis of the Human Genome Diversity Project-Centre d’Etude du Polymorphisme Humain (HGDP-CEPH) panel with 377 microsatellites or short tandem repeats (STR) conducted in five Native Amerindian populations (Pima, Maya, Colombian, Karitiana, and Surui) found a high frequency of a small allele (275 basepairs) at the tetra-nucleotide locus D9S1120, which was absent in 47 other worldwide populations (1). Based on the corresponding number of repeats, this private allele was identified as “9RA” (9 repeats allele). The ubiquitous presence of 9RA in North and South American populations, including the Na-Dene and Aleut-Eskimo, and in related Western Beringian groups suggested that all modern Native American populations originated from the same founding population (2). A later extended survey of 678 STRs in 29 American populations found high frequencies of 9RA across all American regions (average 0.301 in North America, 0.471 in South America, and 0.364 in the full Native American sample), which was also interpreted as evidence of a single main colonization event (3). Interestingly, this interpretation based on a single autosomal marker is in agreement with archeological, mitochondrial, and Y-chromosomal data (4-10). Finally, the single main colonization hypothesis is supported by the following observations: 1) all the chromosomes with 9RA share the same haplotypic background in the vicinity of D9S1120, suggesting they are identical by descent; 2) the positive selection hypothesis was shown as unlikely; and 3) the range of time estimated for the most recent common ancestor for the 9RA marker is consistent with other recent estimates based on archeological and genetic data concerning the origin of Native American populations (11).

The forensic potential of D9S1120 for detecting Native American ancestry was evaluated in a third study, which typed three native and two admixed populations from Colombia and three non-American populations (12). For this purpose, a new primer set reducing the amplicon sizes was designed and an allelic ladder was constructed, characterizing 13 alleles (12). However, the ability of D9S1120 to identify Native American ancestry requires a fuller evaluation in Latin American populations, both for anthropological and forensic genetics purposes. For instance, the greatest part of Mexican population belongs to an ethnic group created by post-Colombian admixture, commonly known as Mestizos (>90%); in Mestizos, the frequency of the European ancestry component increases toward the northwest and the frequency of the Amerindian ancestry component increases toward the central-southeast (13,14). The frequency of African ancestry component is low and evenly distributed through the country (13,14). Mexico has a large number of Amerindian populations, with over 68 ethnic groups representing 9.6% of the total population (15). We analyzed the D9S1120 STR in five Mestizo and eight Amerindian populations from different regions of Mexico. The genetic diversity and population differentiation based on D9S1120 were compared with those obtained by STRs of the Combined DNA Index System (CODIS-STRs).

## Materials and methods

### D9S1120 genotyping

**Figure 1:**
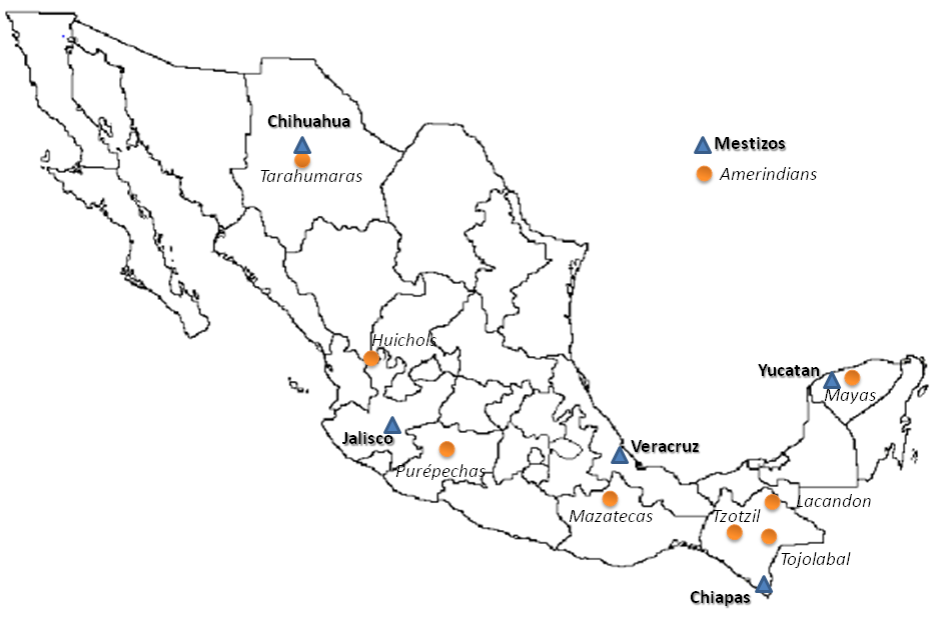
Geographical location of the Mexican populations analyzed in this study: Mestizos (triangles) and Amerindian groups (circles).

DNA was extracted from fresh blood samples by salting-out method (16). Its quality was evaluated by 1% agarose gel electrophoresis and observed by ethidium bromide staining. The study included 247 and 707 unrelated persons from five Mestizo and eight Amerindian populations from Mexico, respectively (Table 1; Figure 1). All participants signed a written informed consent, according to the ethical guidelines of the Helsinki Declaration and the study was approved by the Ethics Research Committee of the CUCiénega, University of Guadalajara. For amplification of D9S1120, we used primers and conditions described by Phillips et al (12). The polymerase chain reaction products were separated by capillary electrophoresis using the ABI Prism 310 and profiles were analyzed with GeneMapper ID software, version 3.2 (Applied Biosystems, Foster City, CA, USA). D9S1120 alleles were named according to the repeat structure and size (bp) described by Phillips et al (12), which follows the International Society of Forensic Genetics guidelines for STR analysis recommending thorough sequence analysis of alleles used to construct reference ladders.

**Table 1:** Description of the Mexican populations analyzed with the short tandem repeat (STR) D9S1120*

Mestizo population	Abbreviation	Location, state	Region	Sample size (n)
**Chihuahua**	Chih	Chihuahua, Chihuahua	North	51
**Jalisco**	Jal	Guadalajara, Ocotlán, Jalisco	West	52
**Veracruz**	Ver	Veracruz, Veracruz	Center	42
**Chiapas**	Chis	Tapachula, Chiapas	Southeast	51
**Yucatán**	Yuc	Mérida, Yucatan	Southeast	51
**Amerindian group**				
**Tarahumara**	Tar	Chihuahua, Chihuahua	North	125
**Huichol**	Hui	San Sebastián Teponamastlán, Jalisco	West	61
**Purépecha**	Pur	Zipiajo and Angahuan, Michoacán	West	111
**Mazateco**	Maz	San Miguel Soyaltepec, Oaxaca	South	41
**Lacandón**	Lac	Lacanjá Chansayab, Ocosingo, Chiapas	Southeast	78
**Tzotzil**	Tzo	San Juan Chamula, Chiapas	Southeast	113
**Tojolabal**	Toj	Las Margaritas, Chiapas	Southeast	52
**Mayas**	May	Yucatan and Quintana Roo	Southeast	126

*For some analyses these populations were grouped to estimate allele and genotype distribution.

### Data analyses

Allele distribution and the following statistical parameters of forensic importance were computed with the PowerStats software (17): observed heterozygosity (Ho), power of exclusion (PE), power of discrimination (PD), polymorphism informativity content (PIC), and typical paternity index (IP). For each population sample, Hardy-Weinberg expectation was verified by exact tests (95% confidence interval, CI) using the Genetic Data Analysis program (GDA), version 1.1 (18). Genetic differentiation was evaluated by F_ST_ distances and exact test *P* values including previously published population data (12) using Arlequin 3.1 software (19). We compared the genetic diversity (*Het*) and population differentiation (F_ST_) based on D9S1120 polymorphism with those previously estimated with nine CODIS-STRs in Mexican Mestizos from Chihuahua, Jalisco, Yucatan, and Veracruz (13,20,21), and available data from Maya and Purépecha Amerindian groups (15). The CODIS-STRs were used for comparison purposes because they were analyzed in all the studied Mexican populations and are included in both Identifiler and Profiler kits (Applied Biosystems). Genetic distances were shown in a multidimensional scaling (MDS) plot to explore the genetic relationships among the populations with SPSS, version 10.0 (SPSS Inc., Chicago, IL, USA). Analysis of molecular variance (AMOVA) was carried out in the total Mexican population sample, and separately in Mestizos and Amerindian groups.

## Results

### Allele frequencies and forensic parameters

In Mexican populations, we identified nine alleles (alleles 9, 10, and 13-19), but the allele 10 was exclusively observed in the Purépecha group. The increased allele number by population was related to the presence of alleles 13 and 19, which were observed in Mestizos from Chihuahua (north), Jalisco (west), and the Maya group (southeast). The smallest allele number was observed in the Tojolobal (4 alleles) and Lacandon (5 alleles) native groups. The modal alleles in Amerindian groups were 9 (38.2%) and 16 (30.5%), but in the Tojolabal and Maya groups the allele 16 was prevalent over the allele 9 (modal values of 47% and 34%, respectively) (Tables 2 and 3). In Mestizos, the modal allele was 16 (39.1%) followed by 9 (21%).

**Table 2:** Statistical parameters of forensic importance in five Mexican-Mestizo populations for the locus D9S1120*^†^

	Chihuahua	Jalisco	Veracruz	Chiapas	Yucatán	Global
Allele	2n = 102	2n = 104	2n = 84	2n = 102	2n = 102	2n = 494
**9**	0.1373	0.2212	0.2381	0.2255	0.2353	0.2105
**13**	0.0098	0.0192	-	-	-	0.0061
**14**	0.0392	0.0288	0.0238	0.0294	0.0294	0.0304
**15**	0.1961	0.0865	0.1429	0.1667	0.0980	0.1376
**16**	0.4118	0.4712	0.2976	0.3627	0.3922	0.3907
**17**	0.1569	0.125	0.2619	0.1667	0.2157	0.1822
**18**	0.0392	0.0385	0.0357	0.0490	0.0196	0.0364
**19**	0.0098	0.0096	-	-	0.0098	0.0061
**MAF**	0.0512	0.0460	0.0584	0.0512	0.0512	
**Genotype**	n = 51	n = 52	n = 42	n = 51	n = 51	n = 247
**9/9**	0.0392	0.0962	0.0952	0.0588	0.0588	0.0691
**9/13**	-	0.0192	-	-	-	0.0041
**9/14**	0.0196	-	0.0238	0.0392	0.0196	0.0203
**9/15**	0.0392	-	0.0714	0.0588	0.0784	0.0488
**9/16**	0.0980	0.1731	0.0476	0.1373	0.1373	0.1219
**9/17**	0.0196	0.0385	0.1429	0.0784	0.0980	0.0732
**9/18**	0.0196	-	-	0.0196	0.0196	0.0122
**9/19**	-	0.0192	-	-	-	0.0041
**13/15**	0.0196	-	-	-	-	0.0041
**13/17**	-	0.0192	-	-	-	0.0041
**14/15**	-	-	-	0.0196	-	0.0041
**14/16**	0.0196	0.0385	0.0238	-	-	0.0163
**14/17**	0.0392	0.0192	-	-	0.0196	0.0163
**14/18**	-	-	-	-	0.0196	0.0041
**15/15**	0.0392	0.0192	0.0238	0.0588	-	0.0285
**15/16**	0.1765	0.0962	0.0952	0.1176	0.0980	0.1138
**15/17**	0.0392	0.0385	0.0714	0.0196	0.0196	0.0366
**15/18**	0.0196	-	-	-	-	0.0041
**15/19**	0.0196	-	-	-	-	0.0041
**16/16**	0.1569	0.2500	0.1429	0.1176	0.1569	0.1667
**16/17**	0.2157	0.0962	0.1190	0.1765	0.2157	0.1667
**16/18**	-	0.0385	0.0238	0.0588	-	0.0244
**16/19**	-	-	-	-	0.0196	0.0041
**17/17**	-	0.0192	0.0714	0.0196	0.0392	0.0285
**17/18**	-	-	0.0476	0.0196	-	0.0122
**18/18**	0.0196	0.0192	-	-	-	0.0081
**Statistical parameters**						Average
**Ho**	0.7451	0.5962	0.6667	0.7451	0.7451	0.6996
**PIC**	0.7116	0.6671	0.7243	0.7220	0.6913	0.7033
**PD**	0.8774	0.8713	0.9048	0.8989	0.8774	0.8860
**PE**	0.5014	0.2863	0.3786	0.5014	0.5014	0.4338
**Typical PI**	1.9615	1.2381	1.5000	1.9615	1.9615	1.7245
***P*^‡^**	0.1027	0.1289	0.5024	0.6445	0.5218	

*Abbreviations: MAF – minimum allele frequency; Ho – heterozygosity observed; PIC – polymorphism informativity content; PD – power of discrimination; PE – power of exclusion; PI – paternity index.

†Modal alleles and genotypes by populations are underlined.

‡Exact test for Hardy-Weinberg expectations.

**Table 3:** Statistical parameters of forensic importance in eight Mexican-Amerindian groups for the locus D9S1120*^†^

	Tarahumara	Huichol	Purépecha	Mazateca	Tzotzil	Tojolabal	Lacandon	Maya	Global
Allele	2n = 250	2n = 122	2n = 222	2n = 82	2n = 226	2n = 104	2n = 156	2n = 252	2n = 1414
**9**	0.44	0.3934	0.4324	0.378	0.354	0.2115	0.5513	0.2659	0.38190
**10**	-	-	0.0045	-	-	-	-	-	0.0007
**13**	-	-	-	-	-	-	-	0.0040	0.0007
**14**	0.004	0.0246	0.0045	0.0122	-	-	-	0.0159	0.0071
**15**	0.132	0.1557	0.1261	0.1585	0.1637	0.0769	0.0705	0.1508	0.1322
**16**	0.324	0.3525	0.2523	0.3415	0.2788	0.4712	0.1603	0.3413	0.3048
**17**	0.088	0.0574	0.1667	0.0732	0.1283	0.2404	0.1603	0.1587	0.1351
**18**	0.012	0.0164	0.0135	0.0366	0.0708	-	0.0577	0.0595	0.0361
**19**	-	-	-	-	0.0044	-	-	0.004	0.0014
**MAF**	0.0204	0.0406	0.0233	0.0578	0.0240	0.0499	0.0325	0.0223	
**Genotype**	n = 125	n = 61	n = 111	n = 41	n = 113	n = 52	n = 78	n = 126	n = 707
**9/9**	0.2080	0.1803	0.1802	0.1951	0.1150	0.0769	0.3077	0.0794	0.1641
**9/10**	-	-	0.0090	-	-	-	-	-	0.0014
**9/14**	0.0080	0.0164	-	-	-	-	-	0.0079	0.0042
**9/15**	0.0960	0.1148	0.1351	0.0488	0.0531	0.0192	0.0769	0.0794	0.0834
**9/16**	0.2720	0.2459	0.2523	0.1707	0.2212	0.1731	0.1410	0.1984	0.2178
**9/17**	0.0880	0.0492	0.0991	0.1220	0.115	0.0769	0.1795	0.0635	0.0976
**9/18**	-	-	0.0090	0.0244	0.0796	-	0.0897	0.0238	0.0297
**9/19**	-	-	-	-	0.0088	-	-	-	0.0014
**13/14**	-	-	-	-	-	-	-	0.0079	0.0014
**14/15**	-	-	0.0090	-	-	-	-	0.0159	0.0042
**14/16**	-	0.0164	-	0.0244	-	-	-	-	0.0028
**14/18**	-	0.0164	-	-	-	-	-	-	0.0014
**15/15**	0.0240	0.0328	0.0180	0.0488	0.0531	-	-	0.0238	0.0255
**15/16**	0.0960	0.0984	0.0450	0.1463	0.1239	0.0962	-	0.1032	0.0863
**15/17**	0.0160	0.0328	0.0270	-	0.0442	0.0385	0.0513	0.0397	0.0325
**15/18**	0.0080	-	-	0.0244	-	-	0.0128	0.0159	0.0071
**16/16**	0.1200	0.1475	0.0721	0.1463	0.0708	0.1731	0.0385	0.0873	0.0976
**16/17**	0.0400	0.0328	0.0541	0.0244	0.0354	0.3269	0.0897	0.1270	0.0820
**16/18**	-	0.0164	0.009	0.0244	0.0354	-	0.0128	0.0714	0.0240
**16/19**	-	-	-	-	-	-	-	0.0079	0.0014
**17/17**	0.0160	-	0.0721	-	0.0265	0.0192	-	0.0397	0.02687
**17/18**	-	-	0.0090	-	0.0088	-	-	0.0079	0.0042
**18/18**	0.0080	-	-	-	0.0088	-	-	-	0.0028
**Statistical parameters**									**Average**
**Ho**	0.6240	0.6393	0.6577	0.6098	0.7257	0.7308	0.6538	0.7698	0.6764
**PIC**	0.6200	0.6380	0.6588	0.6582	0.7083	0.6150	0.5980	0.7237	0.6525
**PD**	0.8393	0.8557	0.8590	0.8673	0.8868	0.8099	0.8268	0.8995	0.8555
**PE**	0.3207	0.3408	0.3658	0.3028	0.4691	0.4774	0.3605	0.5443	0.3977
**Typical PI**	1.3298	1.3864	1.4605	1.2813	1.8226	1.8571	1.4444	2.1724	1.5943
P*	0.0897	0.6252	0.1203	0.2272	0.0364	0.3685	0.1952	0.3071	

*Abbreviations: MAF – minimum allele frequency; Ho – heterozygosity observed; PIC – polymorphism informativity content; PD – power of discrimination; PE – power of exclusion; PI – paternity index.

†Modal alleles and genotypes by populations are underlined.

‡Exact test for Hardy-Weinberg expectations.

**Figure 2:**
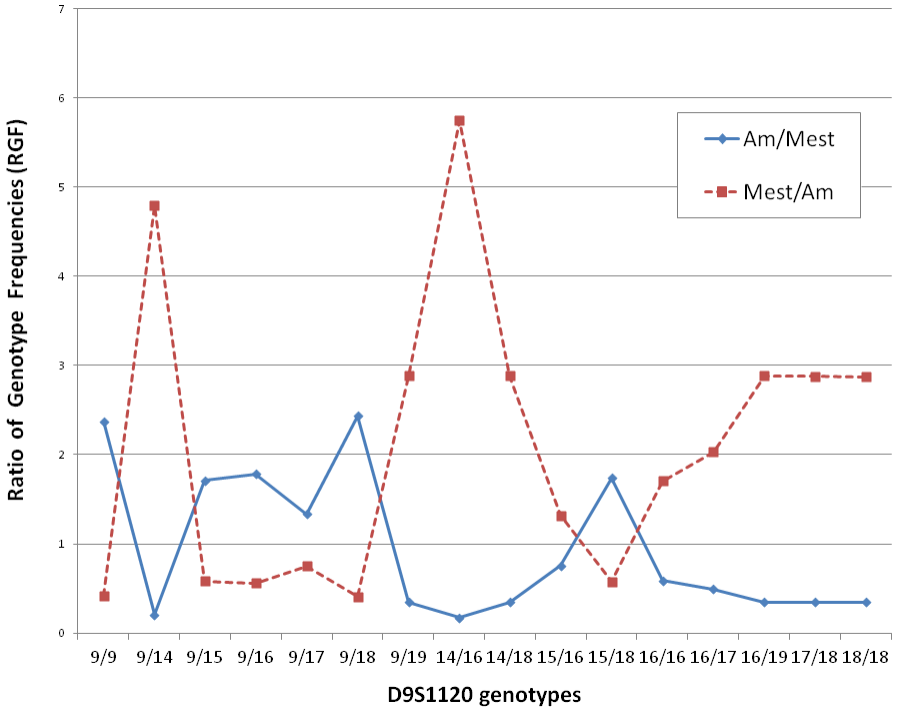
Ratio of genotype frequencies (RGF) plot for D9S1120 between Mexican Ameridians (Am) and Mestizos (Mest), and vice-versa. Genotypes with RGF values below 0.5 were excluded from the plot.

Genotype distributions of D9S1120 were in agreement with the Hardy-Weinberg expectations in all Mexican populations (Table 2 and 3). The only exception was the Tzotzil group, which showed a relatively low *P* value (*P* = 0.0364), however this was not significant after Bonferroni correction. In order to record representative D9S1120 genotypes for the main Mexican population groups, we obtained a ratio of genotype frequencies (RGF) between Mestizos and Amerindians and vice-versa (Figure 2). Although genotypes 9/14 and 14/16 can be used to indicate the Amerindian component of Mestizo origin, their potential may be limited by low population frequencies (≤2.03 and 1.63%, respectively). In general, RGF values indicated the allele 9 was typically observed in Amerindian groups and the alleles 14 and 16 in Mestizos.

### Genetic relatedness between populations

**Figure 3:**
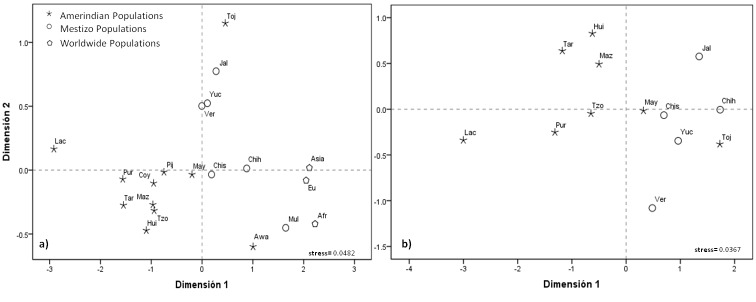
Genetic distances (Fst) represented in multidimensional scaling plots based on D9S1120: (**A**) Mexicans plus reference populations from Europe (Eu), Asia (Asia), Africa (Afr), and from Colombia: Mulaló-Mestizos (Mul), Awa (Awa), Pijao (Pij), and Coyaima (Coy) (12). (**B**) Mexican Amerindian and Mestizo populations (abbreviations are shown in Table 1).

We estimated genetic distances and Fst *P* values based on D9S1120 (Table 4) and represented them graphically (Figure 3). Mexican populations were compared to the reference populations from Europe, Africa, and East Asia (12) (*P* < 0.01) and were clearly separated in the MDS plot (Figure 3A). No differentiation was observed between all five Mexican Mestizo populations but the majority of differences in pairwise comparisons between Mestizo populations and Lacandones, Tarahumaras, Purépechas, and Tzotzils were significant (Table 4). Among native groups, Tojolabales and Mayas showed a high frequency of allele 16 (Table 3), which explains their close relationship with Mexican Mestizos (Figure 3). Similarly, Mulalós from Colombia (12) showed differences from all Mexican Amerindian groups (*P* < 0.01), but were similar to most of Mexican Mestizos, except those from Yucatan (*P* = 0.0032) (data not shown).

**Table 4:** Genetic distances (Fst, below diagonal) and Fst *P* values (above diagonal) between Mexican Mestizos and Amerindian populations*

	Mestizos					Amerindian groups							
	Chih	Jal	Ver	Chis	Yuc	Tar	Hui	Pur	Maz	Tzo	Toj	Lac	May
**Chih**	*******	0.4811	0.1796	0.7476	0.7015	0.0000*	0.0086*	0.0000*	0.0264	0.0000*	0.3464	0.0000*	0.0332
**Jal**	0.0160	*******	0.2024	0.3363	0.2158	0.0000*	0.1468	0.0000*	0.3628	0.0010*	0.1958	0.0000*	0.0389
**Ver**	0.0230	0.0339	*******	0.7544	0.6966	0.0027*	0.0373	0.0163	0.1905	0.0251	0.0552	0.0000	0.2915
**Chis**	0.0072	0.0136	0.0079	*******	0.8938	0.0001*	0.0826	0.0017*	0.3423	0.0879	0.3627	0.0000*	0.9471
**Yuc**	0.0154	0.0104	0.0075	0.0056	*******	0.0098*	0.0359	0.0042*	0.0706	0.0018*	0.8602	0.0014*	0.4635
**Tar**	0.0766	0.0548	0.0515	0.0419	0.0476	*******	0.8436	0.3577	0.3383	0.0031*	0.0000*	0.0000*	0.0000*
**Hui**	0.0547	0.0381	0.0457	0.0285	0.0380	0.0069	*******	0.2822	0.8369	0.1433	0.0009*	0.0001*	0.1848
**Pur**	0.0798	0.0676	0.0350	0.0415	0.0444	0.0135	0.0206	*******	0.0738	0.0290	0.0000*	0.0001*	0.0191
**Maz**	0.0461	0.0331	0.0353	0.0205	0.0306	0.0055	0.0095	0.0161	*******	0.6686	0.0048*	0.0009*	0.2460
**Tzo**	0.0469	0.0438	0.0235	0.0197	0.0302	0.0161	0.0138	0.0139	0.0071	*******	0.0000*	0.0000*	0.1290
**Toj**	0.023	0.0116	0.0245	0.0196	0.0062	0.0724	0.0615	0.0731	0.0545	0.0567	*******	0.0000*	0.1650
**Lac**	0.1593	0.1385	0.0904	0.1052	0.1081	0.0411	0.0609	0.0241	0.0569	0.0470	0.1484	*******	0.0000*
**MY1**	0.0186	0.0193	0.0107	0.0043	0.0101	0.0316	0.0225	0.0309	0.0150	0.0139	0.0266	0.0828	*******

*Significant Fst *P* (<0.01). For abbreviations see Table 1.

A pairwise comparison between Mexican Amerindian groups, including the Mayas (12), showed that the most differentiated populations were Lacandones, Tojolabales, Tarahumaras, Tzotziles, and Purépechas (Table 4). Interestingly, the pooled South American groups including the Awa-Kuaikier, Pijao, and Coyaima from Colombia (12) were similar to all five Mexican Mestizo populations (*P* > 0.01; data not shown) and to the majority of Mexican Amerindian populations, except Lacandones. Individually, the Colombian Awa-Kuaikier population was different from Tarahumaras, Purépechas, Tzotziles, and Lacandones, whereas the Pijao group was different only from Lacandones and Tojolabales. Lacandones was the most distinct and markedly differentiated Mexican Amerindian group (Figure 3B).

### Analysis of molecular variance (AMOVA)

AMOVA based on D9S1120 showed a significant inter-population differentiation among all the Mexican populations (F_ST_ = 4.03%; *P* < 0.0001). However, Amerindians showed larger and significant inter-population differentiation (F_ST_ = 2.81%; *P* < 0.0001) than Mestizos (F_ST_ = 0.44%; *P* = 0.187). When we clustered Mestizos vs Amerindians, the differentiation both between the groups (F_CT_ = 2.19%; *P* = 0.0098) and within the groups was significant (F_SC_ = 1.84%; *P* < 0.000), indicating that the population clustering was not robust. Although differentiation between Amerindians from Mexico and those from Colombia (12) was not significant (F_CT_ = -0.16%; *P* = 0.409), differentiation within the groups was (F_SC_ = 2.25%; *P* < 0.0001). Finally, when population structure was assessed between Amerindians (Mexicans plus Colombians) and populations from Europe, Asia, and Africa (12), differentiation between the groups increased substantially (F_CT_ = 8.54%; *P* < 0.0001).

### Comparison of D9S1120 with CODIS-STRs

**Figure 4:**
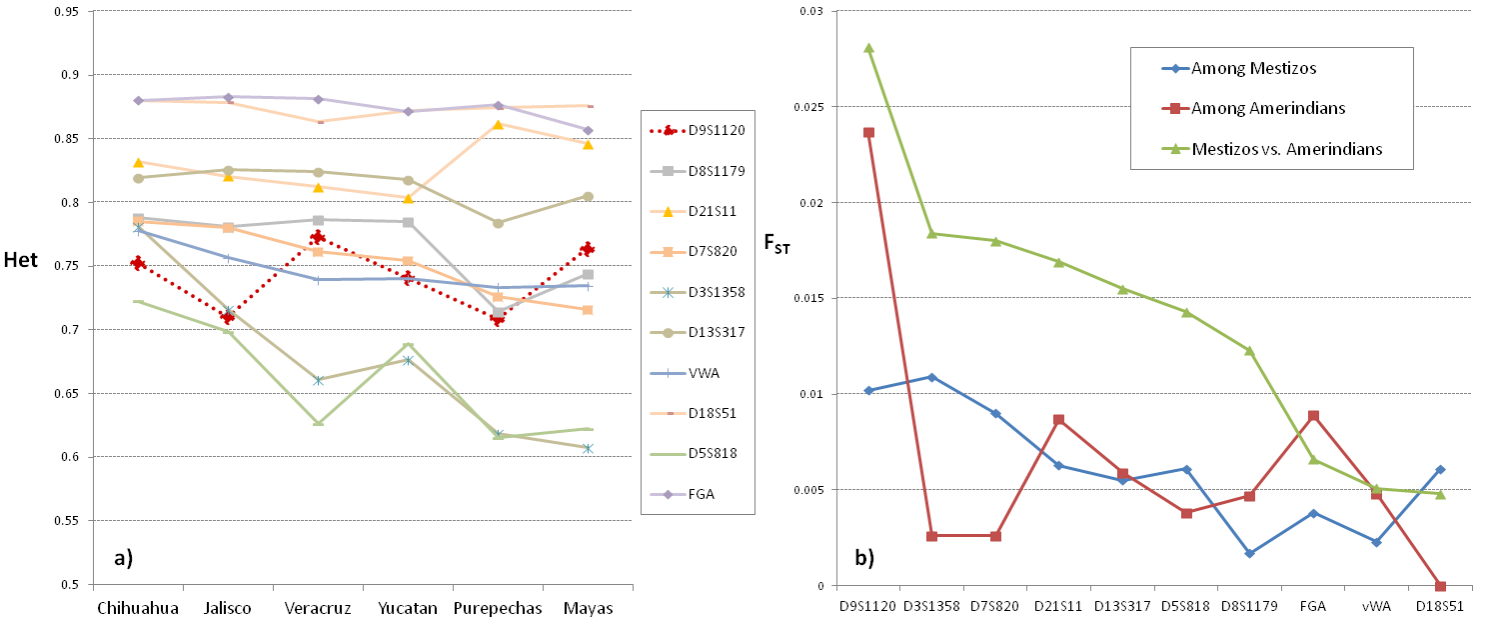
Comparison of the genetic diversity (Het) (**A**) and genetic differentiation (F_ST_) (**B**) based on D9S1120 and 9 CODIS-STRs in 6 Mexican populations (13,15,20,21).

The genetic diversity represented by heterozygosity (Het) and the population differentiation coefficient (F_ST_) based on D9S1120 were compared with those previously obtained using 9 CODIS-STRs from the corresponding Mexican populations (Chihuahua, Jalisco, Veracruz, Yucatan, Purépechas, and the Maya). D9S1120 showed lower genetic diversity than most of the nine CODIS-STRs in most of the populations (Figure 4A), (except D5S818 and D3S1358), but it showed higher diversity in Mestizos from Veracruz and the Mayas than four and five CODIS-STRs, respectively (Figure 4A). D9S1120 had the ability to differentiate Amerindians from Mestizos, and Amerindian groups between each other. However, its ability to differentiate Mexican Mestizos was limited; in fact, it was just a little higher than that of the most of the CODIS-STRs (Figure 4B).

## Discussion

In this study, the STR locus D9S1120 was characterized in Mexican Amerindian groups and the admixed Mestizo population to determine its forensic and anthropological potential; importantly, our findings could apply to other Latin American countries. A total of nine alleles were found in Native Americans and Mestizos, with very similar frequency distributions as in previous reports (2,3,12). Five out of 14 alleles previously described in worldwide surveys were not detected: alleles 11, 12, 17.3, 18.3, and 20; however, these D9S1120 alleles are rare in all populations (11,12). Interestingly, in a previous report including 24 Native American (n = 426) and 53 worldwide populations (n = 1048), the allele 10 was only detected in the Maya, Ojibwa, and Cree populations (3). In our full Mexican Native American sample (n = 1414), the allele 10 was only observed in Purépechas but not in Mayan samples, confirming its very rare frequency, at least among the studied Mexican populations. Another interesting and uncommon allele is the allele 19, which was previously found in the Mayas and whose origin is either Native American or European (12). In our study, the allele 19 (315 bp) was observed in the Tzotzil native group and three Mestizo populations (<1%), with a higher global frequency in Mestizos than in native groups (0.607 vs 0.141%), in line with the observations of Phillips et al (12).

The modal alleles 9RA and 16 displayed a prevalence of 38.2% and 39.1% in Amerindian groups and Mestizos, respectively. Therefore, elevated frequency of allele 9RA and/or low frequency of allele 16 indicate Amerindian ancestry, whereas an opposite pattern indicates an admixture or non-native ancestry (eg, European). In Mestizos, this assumption was evaluated in view of the previously described increasing northwest to southeast gradient of Amerindian ancestry and the opposite gradient of European ancestry. This pattern has been consistently obtained with different genetic systems, such as CODIS-STRs, Y-STRs, and genome-wide single nucleotide polymorphisms analysis (13,14,22). While the allele 16 distribution is in agreement with such ancestry distribution, the 9RA distribution confirms it only partially because its frequency in northern Mestizos from Chihuahua was low (13.7%), and higher in other Mestizo populations (22%-24%).

9RA was prevalent in the majority of Mexican Amerindian groups, except in the Mayas and Tojolobales, where the modal allele was 16 (47.1 and 34.3%, respectively). There are contrasting explanations for such allele distribution. In the Mayas, the large number of alleles and the observed heterozygosity suggests admixture, which is in agreement with previous reports on elevated gene flow (15) and on cultural practices allowing marriage with non-Mayan individuals (23). Conversely, a reduced number of alleles observed in Tojolobales, in addition to their cultural and geographic isolation, suggests genetic drift and/or founder effect. Similarly, Lacandones showed the lowest genetic diversity and largest differentiation from other populations, also suggesting genetic drift effects. This finding, although only based on a single autosomal STR, confirms the conclusions obtained with Y-chromosome markers (Rubi-Castellanos et al, unpublished data 2012) and historical records of geographic and socio-cultural isolation of Lacandones (24,25).

The forensic parameters estimated in this study confirm that D9S1120 can be a useful tool for human identification and molecular anthropology. On average, genetic diversity values (Ho) were larger in Mestizos than Amerindian groups (Table 2 and 3). In most Mexican populations, the informativeness of D9S1120 was lower than that of the majority of CODIS-STRs, largely due to the dominant, high frequencies of 9RA and allele 16. However, given their different distributions (2,3,11,12), D9S1120 potentially allows the discrimination of Amerindian and Mestizo biological samples from those of different origin (eg, European, Asian, or African). We would advocate the use of this STR alongside uni-parental markers (ie, Y-chromosome and mtDNA) and particularly ancestry-informative markers in commercial STR human identification kits to provide the most robust ancestry identification in forensic samples. This would be useful in multi-ethnic countries with a large proportion of Hispanic populations of Amerindian origin (eg, United States).

An interesting finding was that Mestizos and Amerindian groups from Mexico could not be differentiated from their counterparts from Colombia (12), which is in agreement with the hypothesis on the common origin of Native Americans (2,3). Similarly, the population structure and differentiation patterns among some Mexican populations closely agreed with previous analyses based on autosomal STRs (15,26) and Y-chromosome markers (27). Additionally, MDS plot stress-values (stress <0.1) indicated a reasonable reliability of the genetic relationships. In summary, our results emphasize the potential of D9S1120 to differentiate Native American groups from Mestizos and other population groups. Further studies of this unique STR are required, especially in view of its application in the forensic practice.
